# The relationship between mental health literacy and professional psychological help-seeking behavior among Chinese college students: mediating roles of perceived social support and psychological help-seeking stigma

**DOI:** 10.3389/fpsyg.2024.1356435

**Published:** 2024-06-13

**Authors:** Xiao Yang, Jun Hu, Bingren Zhang, Hua Ding, Danying Hu, Hangdong Li

**Affiliations:** ^1^Mental Health Education and Counseling Center, Hangzhou Normal University, Hangzhou, China; ^2^School of Marxism, Hangzhou Normal University, Hangzhou, China; ^3^Affiliated Hospital (School of Clinical Medicine), Hangzhou Normal University, Hangzhou, China; ^4^School of Public Health, Hangzhou Normal University, Hangzhou, China; ^5^Mental Health Education and Counseling Center, Ningbo Polytechnic, Ningbo, China; ^6^Mental Health Education and Counseling Center, China Jiliang University, Hangzhou, China

**Keywords:** professional psychological help-seeking, mental health literacy, psychological help-seeking stigma, perceived social support, college students

## Abstract

**Introduction:**

Mental health literacy is viewed as a significant factor that may facilitate an individual’s pursuit of professional psychological assistance. However, it is important to explore further influencing factors that might underlie this association. This study, employing the framework of the Theory of Planned Behavior (TPB), aims to examine the relationship between mental health literacy and the behavior of seeking professional psychological help, with a focus on the potential mediating roles of perceived stigma and social support in this context.

**Methods:**

We surveyed 911 college students in seven regions of China (406 males and 505 females, aged between 19 and 25 years old; *M*_age_ = 19.65, SD = 1.41) utilizing self-report measures, including the Mental Health Literacy Questionnaire, Professional Psychological Help-Seeking Behavior Scale, Professional Psychological Help-Seeking Stigma Scale, and Perceived Social Support Scale. A chain mediation model was developed to analyze the interconnections between mental health literacy, stigma related to seeking psychological help, perceived social support, and professional psychological help-seeking behaviors.

**Results:**

The mediation effect analysis indicates that: (1) mental health literacy significantly positively correlates with professional psychological help-seeking behaviors; (2) both perceived social support and professional psychological help-seeking stigma significantly mediate the relationship between mental health literacy and professional psychological help-seeking behavior; (3) perceived social support and the stigma associated with seeking professional psychological help play a chained mediating effect between mental health literacy and the behavior of seeking professional psychological help.

**Discussion:**

This study found that mental health literacy indirectly facilitates professional psychological help-seeking behaviors by enhancing the perception of social support and reducing the stigma associated with seeking such help. These findings help in understanding how improving mental health literacy and perceived social support while reducing stigma can increase the likelihood of individuals seeking professional psychological assistance. The results are significant for enhancing the utilization of mental health services and implementing mental health education programs in universities.

## 1 Introduction

Between 2010 and 2020, 13.7% of Chinese university students experienced anxiety, and 20.8% experienced depression (Chen et al., 2022). Similarly, the World Health Organization surveyed first-year college students from 19 universities across eight countries. These studies found that about one-third of the students had at least one common DSM-IV classified disorder, including anxiety, mood, or substance-related conditions. Specifically, the data showed that 35.3% of students had experienced these disorders in their lifetime, with a 31.4% prevalence in the past 12 months (Auerbach et al., 2018). The mental disorders can be treated and, in some cases, prevented (Bienvenu and Ginsburg, 2007; Barrera et al., 2009). Despite the availability of psychological counseling services in universities, over 91% of individuals with mental disorders do not receive adequate treatment (Chen, 2018). While Chinese universities have enhanced mental health services and established counseling centers, many students in need still do not seek effective help, preferring self-reliance initially (Gulliver et al., 2010; Vidourek et al., 2014; Li et al., 2016). Research has identified multiple factors influencing the pursuit of professional psychological help. Preferences for self-management, low perceived need, poor mental health literacy, and financial concerns contribute to a reluctance to seek help (Schnyder et al., 2017; Furnham and Swami, 2018; Shahwan et al., 2020). Stigma, misconceptions about psychiatric treatment, and fear of therapy significantly deter people from seeking help (Jiang and Xia, 2006; Li, 2015; Vidourek and Burbage, 2019). Thus, understanding the factors and internal mechanisms of seeking professional psychological help is vital to improve the use of mental health services in universities and prevent severe outcomes like psychological crises from stress.

### 1.1 Mental health literacy and professional help-seeking behavior

Mental health literacy includes knowledge and concepts aiding in the identification, management, and prevention of mental illnesses, alongside self-help and support skills (Jorm et al., 1997; Jorm, 2012; Ahmad et al., 2021; Galyautdinova et al., 2022). This literacy covers knowledge of mental disorders, attitudes, stigma, positive mental health, seeking psychological help, and efficacy in help-seeking efforts (Bjørnsen et al., 2017; Fu and Zhang, 2018). It reflects the knowledge, attitudes, and behaviors individuals develop to promote personal and others’ mental health and to address mental disorders (Jiang, 2020). Many studies show a positive correlation between mental health literacy and seeking professional psychological help (Jung et al., 2017; Kim et al., 2020; Lindow et al., 2020; Calear et al., 2021; Fung et al., 2021). A systematic review of 53 studies found that 96% showed a strong positive correlation between mental health literacy and young people’s attitudes towards seeking professional psychological help (Radez et al., 2021). Mental health literacy can improve the behavior of seeking professional psychological help (Tomczyk et al., 2018; Gorczynski et al., 2020). However, research findings vary. For instance, short-term interventions for mental health literacy and destigmatization may boost knowledge and reduce stigma (Lanfredi et al., 2019) but not necessarily increase help-seeking behavior (Gulliver et al., 2012a,b; Lindow et al., 2020). Other studies indicate that improving mental health literacy may alter public attitudes towards help-seeking more than the behavior of seeking help itself (Lumaksono et al., 2020).

### 1.2 Stigma of psychological help-seeking and professional psychological help-seeking behavior

Stigma related to mental illness and seeking psychological help is a major barrier to engaging in help-seeking behaviors and accessing mental health services (Vogel et al., 2006; Clement et al., 2015; Kantor et al., 2017; Schnyder et al., 2017). Specifically, stigma around seeking psychological help can result in reluctance, refusal of assistance, and avoidance of psychotherapy (Vogel et al., 2006; Tucker et al., 2013; Lannin et al., 2015). Individuals with mental disorders may avoid seeking help to dodge labels of mental illness and subsequent stereotypes and discrimination (Shechtman et al., 2018; DeLuca et al., 2020). Help-seeking stigma involves derogatory labels for those seeking psychological help, encompassing both public and self-stigma (Hao and Liang, 2011; Michaels et al., 2012; Yu et al., 2022). Public stigma involves negative perceptions from society about seeking psychological help, whereas self-stigma is the internalization of these views by individuals with mental disorders, affecting their willingness to seek help (Wu et al., 2016; Keum et al., 2018; Drury et al., 2022). This study examines the stigma surrounding seeking psychological help.

The Theory of Planned Behavior (TPB) posits that attitudes, subjective norms, and perceived control over actions correlate with individual behavior (Ajzen, 1991). The stigma of seeking psychological help includes emotional and cognitive components, like prejudice against mental illness (Schomerus et al., 2019) and negative views of psychological counseling. This stigma is directly linked to mental health literacy and relates to the pursuit of professional psychological help-seeking behaviors. Thus, the stigma of seeking psychological help could act as a mediator between mental health literacy and professional psychological help-seeking behaviors. Studies show that stigma related to seeking psychological help acts as a mediator between mental health literacy and professional help-seeking behaviors. Many studies find that stigma significantly negatively associates with professional psychological help-seeking behaviors (Wu et al., 2013; Cheng et al., 2018). A systematic review revealed a negative correlation between stigma and mental health help-seeking (Clement et al., 2015). Self-stigma in college students predicts their attitudes towards professional psychological help (Jennings et al., 2015). Another review highlights stigmatization as a major barrier for Chinese adults accessing mental health services (Shi et al., 2020). Conversely, some studies suggest mental health literacy negatively predicts stigma towards psychological help-seeking. A study involving 1,775 Chinese participants showed a negative correlation between mental health literacy and public devaluation and discrimination against those with mental health issues (Yin et al., 2020). Additionally, the stigma’s mediating role between mental health literacy and attitudes towards seeking psychological help was preliminarily confirmed in Korean university students (Kim et al., 2020). Therefore, we hypothesize that stigma towards seeking professional psychological help mediates the relationship between mental health literacy and professional psychological help-seeking behaviors.

### 1.3 Perceived social support and professional psychological help-seeking behavior

Perceived social support, crucial in psychological help-seeking research, involves an individual’s belief in the availability and evaluation of social support (Barrera, 1986). Given the ongoing physical and mental development of college students, their perception of social support plays a crucial role. This sense of being respected and understood is likely intricately connected to their attitudes towards seeking professional psychological help.

Research into the relationship between perceived social support and professional psychological help-seeking behaviors is twofold, with inconsistent findings. Some studies show a significant positive correlation between perceived social support and attitudes towards seeking help, suggesting that ample social support predicts positive attitudes towards professional psychological help (Zheng et al., 2016). Conversely, other studies reveal a negative correlation between social support and mental health service utilization (Snowden, 2007; Maulik et al., 2009). The second aspect concerns the mediating role of perceived social support in buffering against the negative effects of adverse events on attitudes towards professional psychological help-seeking (Huang et al., 2020). Studies suggest that perceived social support negatively correlates with self-stigma in help-seeking and mediates the relationship between self-stigma and seeking professional psychological help (Zhang, 2019). Scholars highlight the moderating role of perceived social support, which diminishes the negative connection of self-stigma with attitudes towards professional psychological help (Xue, 2021).

The inconsistency in research findings highlights the need for further investigation into the relationship between perceived social support and professional psychological help-seeking behaviors. The mediating role of perceived social support between mental health literacy, psychological help-seeking stigma, and professional psychological help-seeking behaviors requires more exploration and validation. Perceived social support directly and negatively affects mental health stigma, which, in turn, reflects the level of mental health literacy (Birtel et al., 2017). Conversely, perceived social support softens the harmful effects of self-stigma on attitudes towards seeking professional psychological assistance (Bu et al., 2023). Thus, perceived social support could serve as a mediator in the relationship between mental health literacy and professional psychological help-seeking behaviors.

### 1.4 Potential chain mediating models

Professional psychological help-seeking is a complex process associated with factors like mental health literacy, supportive social networks, and stigma (Jorm, 2000, 2012; Gulliver et al., 2010; Cornally and McCarthy, 2011; Rüsch et al., 2014; Chen et al., 2020). To understand how mental health literacy works on professional psychological help-seeking behavior, while considering cognitive factors (i.e., perceived social support) and emotional factors (i.e., stigma associated with seeking psychological help), this explanation can draw upon the Theory of Planned Behavior (TPB) (McEachan et al., 2011; da Conceição et al., 2024). The Theory of Planned Behavior (TPB) suggests beliefs shape attitudes, norms, and behavioral control, which predict intentions, ultimately driving behaviors (Fishbein and Ajzen, 2010). Within the TPB framework, an individual’s behavioral intentions and perceived behavioral control together may shape the final behavior. One finding emphasized the significance of both attitude and perceived behavioral control in forecasting the intentions and actions of Chinese college students regarding help-seeking, and highlighted a discrepancy between their intentions and actual behaviors in seeking help (Wang et al., 2023). Another study indicated that attitudes were the strongest determinant of intentions to seek help, with coping effectiveness and stigma also playing significant roles. A Theory of Planned Behavior (TPB)-based model was shown to effectively predict the intentions and actions of college students with psychological issues in seeking professional assistance (Pan and Hao, 2023). In this context, mental health literacy can indirectly facilitate the pursuit of professional psychological help by boosting perceived social support and mitigating the stigma linked to seeking such help. Reducing stigma via perceived social support enhances individuals’ perceived behavioral control, thus potentially increasing the likelihood of action towards seeking professional psychological help. This elucidates the pivotal role of mental health literacy in augmenting social support perceptions and diminishing stigma, ultimately possibly empowering individuals to seek the professional help they need. Thus, perceived social support and the stigma associated with professional psychological help-seeking may play a chained mediating role between mental health literacy and professional psychological help-seeking behavior.

### 1.5 The current study

**Figure 1:**
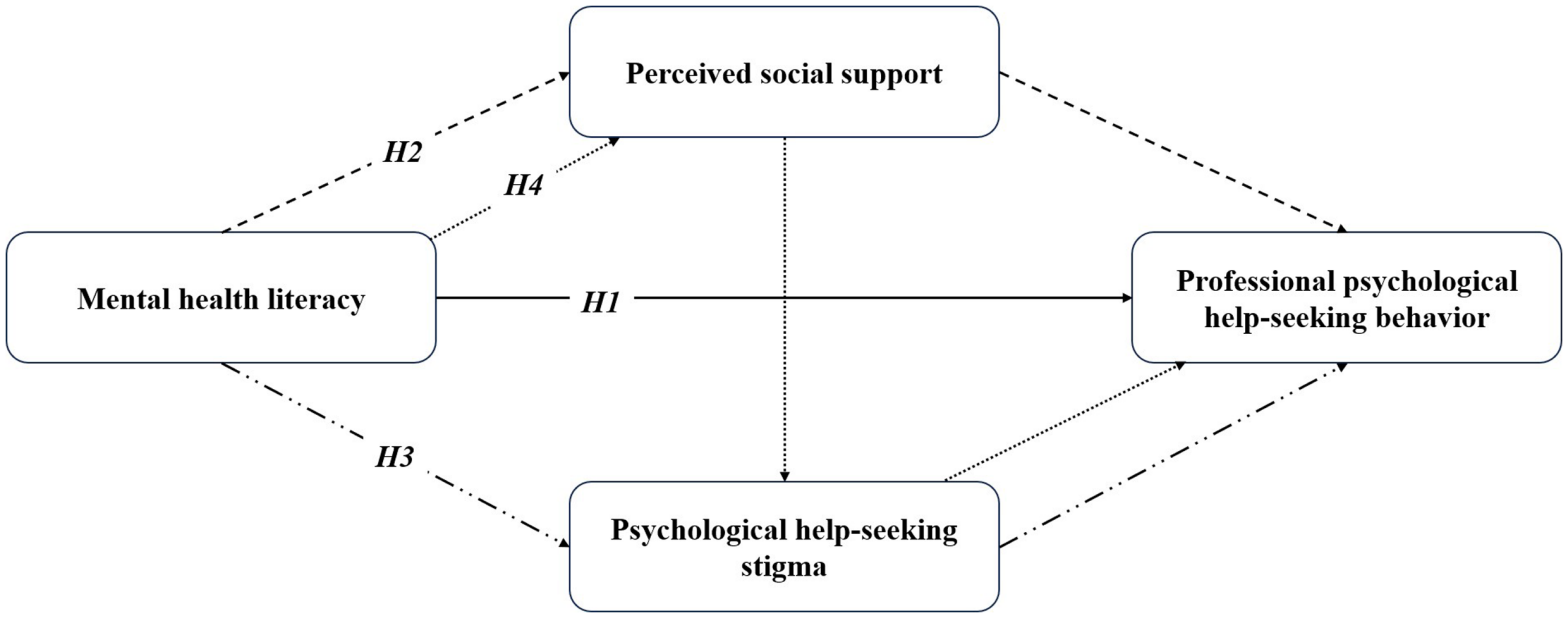
The hypothesized model of mental health literacy, psychological help-seeking stigma, perceived social support, and professional psychological help-seeking behavior.

Mental health literacy, linked to psychological help-seeking stigma, perceived social support, and help-seeking behaviors, needs more research to fully understand its relationship with help-seeking behaviors. This study aims to explore how mental health literacy, psychological help-seeking stigma, and perceived social support correlate with professional help-seeking behaviors, focusing on the mediating roles of stigma and social support in this relationship. Given these established connections above, we gathered data from Chinese university students to analyze a model delineating how mental health literacy link to professional psychological help-seeking behaviors, mediated by stigma and perceived social support. More specifically, we propose the following research hypotheses (refer to Figure 1):

*H1*: Mental health literacy significantly positively correlates with professional psychological help-seeking behaviors;

*H2*: Perceived social support significantly mediates the relationship between mental health literacy and professional psychological help-seeking behaviors;

*H3*: Professional psychological help-seeking stigma significantly mediates the relationship between mental health literacy and professional psychological help-seeking behaviors;

*H4*: Perceived social support and the stigma associated with seeking professional psychological help play a chained mediating effect between mental health literacy and the behavior of seeking professional psychological help.

## 2 Methods

### 2.1 Participants

We performed a cross-sectional study involving Chinese college students, randomly sampled from seven regions across China. After providing informed consent, participants completed the questionnaire online. Initially, the study involved 1,178 university students. After excluding for partial responses, completing the survey in less than 30 s, or uniform responses across items, the final sample size was 911 (406 males and 505 females, aged between 19 and 25 years old; *M*_age_ = 19.65, SD = 1.41), yielding a 77.33% response rate. The Research Ethics Committee of Hangzhou Normal University approved this study.

Table 1 displays the demographic characteristics of the 911 participants (505 females, 406 males), including freshmen through seniors and graduate students. Of the study’s participants, 80 individuals (8.8%) reported seeking professional psychological counseling services.

**Table 1:** Descriptive of socio-demographic (*N* = 911).

	Total *N* (Percentage)
**Gender**	
Male	406 (44.6)
Female	505 (55.4)
**Grade**	
Freshman (1st year)	667 (73.2)
Sophomore (2nd year)	128 (14.1)
Junior (3rd year)	15 (1.6)
Senior (4th year)	34 (3.7)
Graduate and above	67 (7.4)
**Seek Professional Psychological Help**	
Yes	80 (8.8)
No	831 (91.2)

### 2.2 Measures

#### 2.2.1 Mental health literacy

Mental health literacy was evaluated with the Chinese Mental Health Literacy Scale (MHLS) developed by Ming et al. (2021), based on Jung et al.’s original version (Jung et al., 2016). The scale comprises three subscales—mental health knowledge (with 12 items, e.g., “Psychological counseling is an effective method to treat depression”), beliefs (with 10 items, e.g., “Recovery from mental illness primarily depends on luck or fate”), and resources (with 4 items, e.g., “I know where to get mental health services such as on-campus psychological counseling”)—for a total of 26 items. Participants rated their agreement on a six-point scale from 1 (strongly disagree) to 5 (strongly agree), where 6 indicated a lack of knowledge on the item. Scores for knowledge are based on positive responses, for beliefs on reverse scoring, and for resources on a dichotomous scale (0 for ‘do not know’, 1 for others), with higher scores indicating greater mental health literacy. The Chinese MHLS has demonstrated good reliability and validity (Ming et al., 2021). In this study, the Chinese MHLS showed excellent internal consistency (Cronbach’s *α* = 0.951). The subscales for knowledge, beliefs, and resources recorded Cronbach’s *α* coefficients of 0.970, 0.977, and 0.937, respectively, indicating acceptable reliability. Furthermore, confirmatory factor analysis affirmed the scale’s model fit: *χ*^2^/df = 3.027, CFI = 0.978, TLI = 0.976, SRMR = 0.028, RMSEA = 0.047.

#### 2.2.2 Professional psychological help-seeking behavior

The assessment of professional psychological help-seeking behavior is conducted using the Professional Psychological Help-Seeking Scale. Inspired by Wu et al.’s (2011) research, Weng (2021) revised and developed the scale. Wu et al. focused the scale’s measurements on attitudes towards mobile healthcare applications, perceived behavioral control, subjective norms, and behavioral intentions. This was aimed at evaluating medical professionals’ psychological attitudes and perceptions related to adopting mobile health technology, to understand and predict their usage behavior. Weng adapted Wu et al.’s scale by substituting “the use of mobile devices in wireless healthcare” with “professional psychological help-seeking behavior,” localized the scale with revisions, and confirmed its reliability with a Cronbach’s *α* of 0.93, denoting high reliability (Weng, 2021).

The scale includes subscales for measuring attitudes towards psychological counseling (with 4 items, e.g., “Seeking psychological counseling is a good idea”), subjective norms (with 3 items, e.g., “People important to me such as family and close friends would think: Under necessary conditions, I should seek psychological counseling”), perceived behavioral control (with 3 items, e.g., “I have sufficient resources, knowledge, and ability to undergo psychological counseling”), and behavioral intention (with 3 items, e.g., “When I have access to psychological counseling, I am inclined to experience it”), comprising a total of 13 items rated on a five-point Likert scale. Participants rated their agreement with each item on a scale from 1 (strongly disagree) to 5 (strongly agree), where higher scores suggest a higher propensity for seeking professional psychological help. The scale showed good internal consistency in this study, with a Cronbach’s *α* of 0.884. The subscales for psychological counseling attitudes, subjective norms, perceived behavioral control, and behavioral intentions reported Cronbach’s alpha coefficients of 0.931, 0.922, 0.789, and 0.925, respectively, indicating they all fall within acceptable ranges. Additionally, confirmatory factor analysis confirmed the scale’s model fit, evidenced by indices: *χ*^2^/df = 2.028, CFI = 0.993, TLI = 0.991, SRMR = 0.026, RMSEA = 0.034.

#### 2.2.3 Psychological help-seeking stigma

The Stigma for Seeking Professional Psychological Help Scale (SSPPH) assessed participants’ stigma towards seeking psychological help. Revised by Hao and Liang (2011) from the Stigma Scale for Receiving Psychological Help (SSRPH) by Komiya et al. (2000) and the Self-Stigma of Seeking Help Scale (SSOSH) by Vogel et al. (2006), the scale measures public and self-stigma, each with 5 items (e.g., “It is best for one to hide from others that they have seen a psychologist” “Seeking psychological help would make me feel less intelligent”). Participants rated each item on a 5-point scale from 1 (strongly disagree) to 5 (strongly agree), where higher scores signify greater stigma towards seeking professional psychological help. The Chinese version of the SSPPH has shown good reliability and validity (Hao and Liang, 2011). In this study, the SSPPH showed good internal consistency (Cronbach’s *α* = 0.892), with self-stigma and public stigma subscales recording Cronbach’s *α* of 0.888 and 0.893, respectively, indicating acceptable reliability. Additionally, confirmatory factor analysis affirmed the scale’s model fit: *χ*^2^/df = 2.909, CFI = 0.988, TLI = 0.983, SRMR = 0.024, RMSEA = 0.046.

#### 2.2.4 Perceived social support

The Perceived Social Support Scale (PSSS) was utilized to evaluate participants’ perceived social support. This study’s PSSS, revised from the original scale by Blumenthal et al. (1987), was developed by Jiang (1999). It comprises subscales for family support, friend support, and other support, each with 4 items (e.g., “My family is capable of offering me tangible and concrete help” “When faced with challenges, I can depend on my friends” “I can share joys and sorrows with some people”), making a total of 12 items rated on a seven-point scale. Participants rated their agreement with each item on a scale from 1 (strongly disagree) to 7 (strongly agree), where higher scores reflect greater perceived social support. The PSSS has demonstrated strong reliability and validity (Jiang, 1999). The PSSS showed excellent internal consistency in this study (Cronbach’s *α* = 0.925), with Cronbach’s *α* for the family support, friend support, and other support subscales being 0.921, 0.904, and 0.921, respectively, indicating reliable measurement. Furthermore, confirmatory factor analysis confirmed the scale’s model fit indices: *χ*^2^/df = 2.246, CFI = 0.992, TLI = 0.990, SRMR = 0.036, RMSEA = 0.037.

### 2.3 Procedure

The survey was conducted online via the Wenjuanxing platform—a professional service for surveys, exams, assessments, and voting. It specializes in offering users powerful, user-friendly tools for designing questionnaires, collecting data, creating custom reports, and analyzing survey results. Initially, researchers designed the survey, input, and edited content on Wenjuanxing, generating a QR code for the questionnaire. Subsequently, researchers shared the QR code with students through DingTalk, explaining the informed consent process and questionnaire completion. After consenting, participants scanned the QR code to access the questionnaire, starting with an online informed consent form that detailed the study’s purpose, confidentiality measures, and participant rights. Upon agreement, they proceeded to the survey, beginning with demographic questions followed by four subsequent questionnaires. Completing the survey took about 20 min. Participants could exit the survey at any time, and a sincere thank you was displayed upon completion. After removing invalid responses, 911 questionnaires were analyzed.

### 2.4 Data analysis

Internal consistency, descriptive statistics, and correlational analyses were conducted with SPSS 25.0, and model fit and mediation effects were assessed using AMOS 24.0. Descriptive analysis, internal consistency checks, and Pearson’s correlation analysis were initially performed using SPSS 25.0. AMOS 24.0 was then used to assess the serial mediation of professional psychological help-seeking stigma and perceived social support between mental health literacy and professional psychological help-seeking behavior, and to evaluate the structural equation model’s fit. Results showed a CMIN/DF (chi-square to degrees of freedom ratio) of 4.317 and an RMSEA of 0.060, both within acceptable limits, indicating a reliable and applicable model. Other fit indices, including the IFI, CFI, and TLI, ranged from 0.9 to 1, demonstrating good structural validity and fit of the model.

## 3 Results

### 3.1 Preliminary and correlation analyses

Exploratory factor analysis in SPSS 25.0, using Harman’s single factor test, identified 12 factors with eigenvalues greater than 1. The first common factor explained 34.76% of the variance, below the 40% threshold, indicating minimal common method bias. Therefore, common method bias is not a significant concern in this study. Descriptive statistics and Pearson correlation analyses focused on mental health literacy, professional psychological help-seeking stigma, perceived social support, and help-seeking behaviors. Findings revealed significant correlations between mental health literacy, professional psychological help-seeking stigma, perceived social support, and help-seeking behaviors. Mental health literacy negatively correlated with stigma (*r* = −0.485, *p* < 0.01) and positively with help-seeking behaviors (*r* = 0.597, *p* < 0.01) and social support (*r* = 0.392, *p* < 0.01). Stigma negatively correlated with help-seeking behaviors (*r* = −0.636, *p* < 0.01) and social support (*r* = −0.533, *p* < 0.01), while social support positively correlated with help-seeking behaviors (*r* = 0.615, *p* < 0.01) (Tables 2, 3).

**Table 2:** Average scores and overall averages for all variable dimensions.

Dimensions	Dimensional average M ± SD	Total average M ± SD
**Mental Health Literacy**		
Mental health knowledge	3.680 ± 0.869	44.165 ± 10.424
Beliefs	2.854 ± 1.113	28.536 ± 11.131
Resources	3.574 ± 0.825	14.293 ± 3.297
**Professional Psychological Help-seeking Behavior**		
Attitudes Towards Psychological Counseling	3.795 ± 0.936	15.179 ± 3.745
Subjective Norms	3.674 ± 0.845	11.023 ± 2.534
Perceived Behavioral Control	3.654 ± 0.700	10.962 ± 2.098
Behavioral Intention	3.717 ± 0.858	11.150 ± 2.574
**Stigma of Seeking Professional Psychological Help**		
Self-stigma	2.545 ± 0.791	12.722 ± 3.956
Public Stigma	2.392 ± 0.822	11.961 ± 4.109
**Perceived Social Support**		
Family	5.049 ± 1.352	20.195 ± 5.407
Friend	5.247 ± 1.189	20.988 ± 4.756
Other	5.007 ± 1.267	20.027 ± 5.070

**Table 3:** Descriptive statistics and correlation analysis of all variables.

	M ± SD	1	2	3	4
1. Mental Health Literacy	101.270 ± 19.104	–			
2. Stigma of Seeking Professional Psychological Help	24.683 ± 6.886	−0.485**	–		
3. Perceived Social Support	61.211 ± 12.622	0.392**	−0.533**	–	
4. Professional Psychological Help-seeking Behavior	11.150 ± 2.574	0.597**	−0.636**	0.615**	–

*N* = 911, *p < 0.05, **p < 0.01, and ***p < 0.001.

### 3.2 Mediation analyses

Structural equation modeling analyzed theoretical hypotheses, using mental health literacy as the predictor, perceived social support and professional psychological help-seeking stigma as mediators, and attitudes and behaviors towards psychological help as outcomes. Using Amos 24.0, the model’s fit was assessed, yielding *χ*^2^/df = 4.317, RMSEA = 0.060, IFI = 0.951, CFI = 0.963, TLI = 0.951. These indices suggest the model exhibits a strong fit, rendering the mediation model acceptable (see Table 4).

**Table 4:** Structural equation model fit index.

	*χ*^2^/df	IFI	GFI	TLI	RMSEA
Model	4.317	0.951	0.963	0.951	0.060

**Figure 2:**
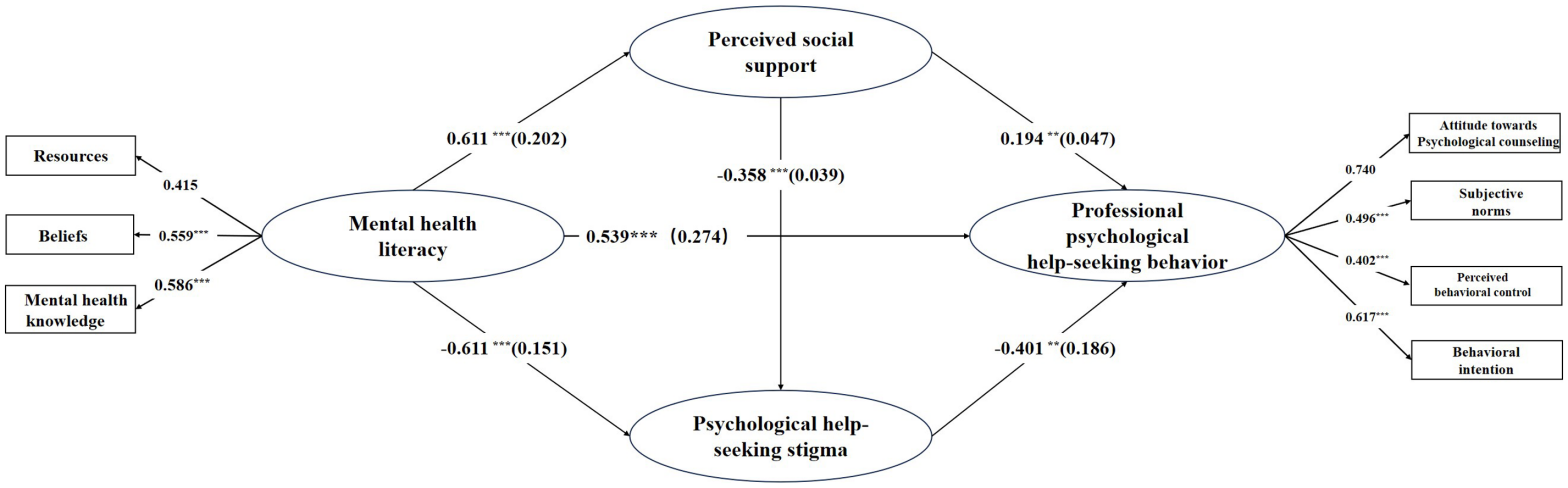
The final chain mediation model. *p < 0.05, **p < 0.01, and ***p < 0.001. Controlling for gender, age, and grade.

After controlling for gender, age and grade, mediation analysis (depicted in Figure 2 and Table 5) reveals significant direct effects: mental health literacy positively associates with perceived social support (*β* = 0.61, *p* < 0.001) and negatively links to professional psychological help-seeking stigma (*β* = −0.61, *p* < 0.001). It also positively affects professional psychological help-seeking behavior (*β* = 0.54, *p* < 0.001), whereas professional psychological help-seeking stigma negatively correlates with this behavior (*β* = −0.40, *p* < 0.001). Additionally, perceived social support positively affects professional psychological help-seeking behavior (*β* = 0.19, *p* < 0.001). The mediation effects were assessed using the bias-corrected nonparametric percentile Bootstrap method. The 95% Bootstrap confidence intervals for the mediation effects do not include 0, demonstrating that professional psychological help-seeking stigma and perceived social support significantly mediate the relationship between mental health literacy and help-seeking behavior. The mediation effects are manifested through three distinct paths:

1. Mental health literacy → perceived social support → professional psychological help-seeking behavior;
2. Mental health literacy → professional psychological help-seeking stigma → professional psychological help-seeking behavior;
3. Mental health literacy → perceived social support → professional psychological help-seeking stigma → professional psychological help-seeking behavior.

**Table 5:** Chain mediation model.

Pathway	*β*	SE	Boot LLCI	Boot ULCI
Total effect	0.498	0.022	0.455	0.542
Direct effect	0.267	0.021	0.226	0.308
Indirect effect	0.231	0.019	0.196	0.267
Mental health literacy → Perceived social support → Professional psychological help-seeking behavior	0.130	0.015	0.160	0.102
Mental health literacy → Psychological help-seeking stigma → Professional psychological help-seeking behavior	0.147	0.016	0.178	0.117
Mental health literacy → Perceived social support → Psychological help-seeking stigma → Professional psychological help-seeking behavior	0.043	0.007	0.057	0.031

*β*, standardized regression coefficient; SE, standard error; LCI, lower bound of 95% confidence interval; UCI, upper bound of 95% confidence interval.

The indirect effect of mental health literacy on professional psychological help-seeking behavior via perceived social support was significant (*β* = 0.130, 95% CI = 0.102 to 0.160), contributing to 39.98% of the relative mediating effect. Similarly, the indirect effect via stigma associated with seeking professional psychological help was significant (*β* = 0.147, 95% CI = 0.117 to 0.178), accounting for 45.26% of the relative mediating effect. Additionally, the sequential indirect effect through both perceived social support and stigma was significant (*β* = 0.043, 95% CI = 0.031 to 0.057), with a relative mediating effect of 13.16%.

## 4 Discussion

This study, focusing on mental health literacy and college students’ propensity to seek professional psychological help, examines the combined effects of mental health literacy, the stigma of seeking psychological help, perceived social support on this propensity, leading to the following main conclusions:

1. Mental health literacy significantly positively correlates with professional psychological help-seeking behaviors;
2. Perceived social support significantly mediates the association between mental health literacy and the propensity to seek professional psychological help;
3. The stigma of seeking professional psychological help strongly mediates the relationship between mental health literacy and the inclination to seek help;
4. Perceived social support and the stigma of seeking professional psychological help are significantly associated in a chained mediation with how mental health literacy relates to the inclination to seek help.

### 4.1 The direct effect of mental health literacy on seeking professional psychological help

In line with our hypothesis, we found a direct link between mental health literacy and professional psychological help-seeking behavior, aligning with previous findings (Tomczyk et al., 2018; Ratnayake and Hyde, 2019; Gorczynski et al., 2020; Kim et al., 2020; Lindow et al., 2020; Calear et al., 2021; Fung et al., 2021; Radez et al., 2021). Enhanced mental health literacy is associated with improved help-seeking behavior. Students possessing higher levels of mental health literacy demonstrate an enhanced capability to recognize mental disorders and a more pronounced tendency to pursue professional psychological assistance (Gorczynski et al., 2017; Rafal et al., 2018). Consequently, this increases the probability of seeking assistance for mental health concerns via personal or alternative approaches (Xiao and Yang, 1987). This finding can be attributed to two main factors: First, understanding mental health and psychological disorders significantly enhances awareness of crucial psychological symptoms, prompting individuals to proactively seek professional assistance (Goldney et al., 2002; Jorm and Kelly, 2007; Siegenthaler et al., 2012; Fung et al., 2021). Second, proficiency in mental health knowledge diminishes prejudice and discrimination against mental illness (Thornicroft et al., 2007; Nemec et al., 2015; Clough et al., 2018; Fang et al., 2020). This, in turn, probably fosters an objective perspective towards psychological counseling and treatment and reduces the reluctance and stigma associated with seeking help for psychological disorders.

### 4.2 The mediation effect of the stigma related to professional psychological help

The study suggested that the stigma linked to seeking professional psychological assistance acts as a mediator between mental health literacy and the behavior of seeking such help, in line with prior studies (Vogel et al., 2006; Clement et al., 2015; Schnyder et al., 2017; Shechtman et al., 2018; DeLuca et al., 2020). First, mental health literacy significantly predicts lower stigma towards seeking professional psychological help and enhancing literacy levels can mitigate this stigma (Shi et al., 2020; Yin et al., 2020). Second, the stigma tied to seeking professional psychological help negatively associates with behavior, where both public and self-stigma are harmful, as shown in previous studies (Wu et al., 2013; Cheng et al., 2018). Self-stigma regarding seeking psychological help stems from broader public stigma. In highly stigmatized environments, this external stigma is often internalized, leading to avoidance and rejection of professional help (Brenner et al., 2019). Among college students, unfamiliarity with mental health resources and perceptions of insufficient time are recognized barriers to seeking professional assistance (Czyz et al., 2013; Marsh and Wilcoxon, 2015; Jennings et al., 2017; Lui et al., 2022).

### 4.3 The mediation effect of perceived social support

This study discovered that perceived social support serves as a mediator between mental health literacy and the pursuit of professional psychological assistance. This aligns with prior studies highlighting the positive association of knowledge and beliefs with mental disorders, along with social support, on the pursuit of mental health assistance (Eisenberg et al., 2007; Woodward et al., 2008; Lakey and Orehek, 2011; Downs and Eisenberg, 2012). First, mental health literacy is a strong predictor of perceived social support, encompassing the knowledge and concepts essential for identifying, managing, and preventing mental disorders, along with self-help and aiding others (Jorm et al., 1997; Jorm, 2012). A high level of mental health literacy fosters greater perceived social support, enhancing individuals’ confidence that psychological issues are supportable and understandable through increased awareness and access to assistance channels. Second, perceived social support significantly positively predicts professional psychological help-seeking behavior, consistent with some research findings: higher levels of perceived social support are related to more positive attitudes towards seeking help (Miville and Constantine, 2006; Koydemir-Özden, 2010; Çebi and Demir, 2019). Enhancing knowledge about mental health and disorders fosters more positive attitudes towards these conditions, aiding college students in maintaining their and others’ mental well-being, managing mental disorders, and supporting others (Puspitasari et al., 2020). Through this process, individuals feel more supported and understood, reducing the stigma associated with self-help and aiding others with mental disorders, thereby encouraging professional psychological help-seeking behavior.

### 4.4 The chain mediating models

The correlation between mental health literacy and seeking professional psychological assistance can be further understood through the chain mediating effect of stigma related to seeking professional help and perceived social support, in alignment with the initial hypothesis.

First, mental health literacy indirectly facilitates professional psychological help-seeking behaviors by enhancing the perception of social support and alleviating the stigma associated with seeking professional psychological assistance. Higher mental health literacy often positively links to a better understanding of mental health’s significance and the need for professional assistance, fostering a positive attitude towards seeking help (Lauber et al., 2003; Tomczyk et al., 2018; Lumaksono et al., 2020). This positive attitude could prompt more open discussions about mental health, enhancing the perception of social support. Perceived social support reflects the attitude that seeking help is both accepted and supported. Consequently, perceiving high social support may lead individuals to believe their social network, like family and friends, supports their decision to seek professional psychological assistance (Kawachi and Berkman, 2001; Thoits, 2011). This perception acts as a positive social pressure, encouraging actions like seeking psychological help and reducing fears associated with social stigma (Chang et al., 2021). Perceived social support can mirror actual support and resources available to an individual, where higher levels of perceived support foster courage and confidence to tackle challenges, enhance support from interpersonal relationships, motivate help-seeking behaviors, and lessen the effects of self and public stigma on seeking professional psychological help, thereby increasing the likelihood of seeking such assistance.

Second, mental health literacy enhances the likelihood of engaging in professional psychological help-seeking behaviors by fostering the perception of social support and strengthening individuals’ perceived behavioral control. Stigma linked to seeking professional psychological help can diminish individuals’ perceived behavioral control, making them feel unable to seek help due to fears of being stigmatized or receiving negative judgments (Barney et al., 2006; Eisenberg et al., 2009; Schomerus et al., 2012; Cheng et al., 2013). However, if the impact of stigma is mitigated through perceived social support, individuals might feel they have greater capacity to overcome these barriers, thereby enhancing perceived behavioral control. As previously stated, perceived social support can counteract the negative effects of stigma on seeking professional psychological help (Birtel et al., 2017; Bu et al., 2023), affirming the protective role of perceived social support (Zhang, 2019; Xue, 2021).

Thus, this study discovers that perceived social support and the stigma associated with seeking professional psychological help play a chain-mediated role between mental health literacy and seeking professional psychological assistance. Mental health literacy plays a crucial role in enhancing the concept of social support and reducing stigma, ultimately enabling individuals to seek the professional help they need. This series of interactive processes collectively foster an individual’s tendency to seek and accept professional psychological help when faced with mental distress.

## 5 Conclusion

Using path analysis, the study indicates a positive association between mental health literacy and the tendency to seek professional psychological help. Additionally, it examined the sequential mediation of perceived social support and stigma in the context of mental health literacy and the stigma of seeking professional psychological help. This offers insights to encourage proactive engagement in seeking psychological assistance among college students, to enhance the use of mental health services, and to support their psychological well-being.

Based on these findings, two primary strategies are recommended: first, to deepen students’ understanding of mental disorders and psychological counseling services through mental health education; second, to encourage proactive help-seeking behavior. This helps to create a clear practical framework for university mental health education programs. Given that mental health literacy is significantly associated with reduced stigma in seeking professional psychological help and recognizing the harmful effects of public and self-stigma, universities are encouraged to improve students’ understanding of mental disorders and psychological counseling services (Nutbeam, 2000; Wright et al., 2006; Kelly et al., 2007; Reavley et al., 2012). A finding indicated that a brief intervention to counteract stigma corresponded with improved attitudes toward seeking help among university students, with variations observed among specific subgroups (Shahwan et al., 2020). This strategy has the potential to reduce public stigma, prevent its transformation into self-stigma, lower student resistance to psychological support, and boost their willingness to independently seek professional psychological assistance.

Furthermore, the research indicates that enhancing perceived social support may reduce the stigma’s negative effects on attitudes towards professional psychological help (Wu et al., 2013; Cheng et al., 2018). University mental health programs can also capitalize on this by strengthening the protective role of perceived social support in seeking professional psychological help. Schools are encouraged to enhance familial emotional support through parenting activities, develop peer support platforms, and provide comprehensive psychological services to further bolster school support. This has the potential to significantly improve the level of social support available to university students. Research has indicated that training focused on resources enhances how university students perceive and utilize social support, which decreases issues related to mental health and bolsters their strategies for coping (Schmiedl and Kauffeld, 2023). Moreover, enhancing students’ awareness of their social support can help to reduce oversights or misunderstandings, utilizing the regulatory effect of perceived social support in seeking professional psychological help and thus promoting such behavior. This approach emphasizes the importance of integrated strategies in university mental health education to better facilitate students’ psychological well-being and engagement with mental health services. For instance, a described project initiates a diverse plan that begins with educating Gatekeeper volunteers to promptly identify and refer students in need. It also includes the creation of a Student Observatory for continuous online symptom monitoring and implements a layered psychological support strategy. This program spans from initial digital self-help tools to individual therapy sessions, aiming to establish a thorough support network for university students’ mental health (Torres et al., 2023).

There are two primary limitations in this study. First, the cross-sectional study design constrains the research to depict the psychological help-seeking behavior and its influencing factors of the sample group at a specific time point, permitting only a limited interpretation of the correlations between variables and not the dynamic changes in the group’s behavior. Therefore, the study’s results are constrained in terms of causal inference. Second, this research faces limitations regarding the diversity of the sample’s geographic origins. Future studies may benefit from incorporating longitudinal tracking of the samples, thereby enabling the observation of the temporal dynamics of the variables. Additionally, exploring other potential influencing factors and their interplay could furtherly make the study of determinants of help-seeking behavior more comprehensive. Future studies might consider including a wider variety of cities in selecting participants’ birthplaces as well. Doing so would assist in comparing the psychological help-seeking behavior of samples from diverse areas and examining how regional cultural and economic factors connect with such behavior, thus enhancing the universal applicability of the research results.

## Data availability statement

The original contributions presented in the study are included in the article/supplementary material, further inquiries can be directed to the corresponding author.

## Ethics statement

The studies involving humans were approved by Hangzhou Normal University Academic Ethics and Academic Morality Committee. The studies were conducted in accordance with the local legislation and institutional requirements. The participants provided their written informed consent to participate in this study.

## Author contributions

XY: Writing – review & editing, Data curation, Writing – original draft, Methodology, Formal analysis, Conceptualization. JH: Data curation, Writing – review & editing, Supervision, Resources, Funding acquisition, Formal analysis. BZ: Supervision, Writing – review & editing. HD: Writing – review & editing, Data curation. DH: Writing – review & editing, Data curation. HL: Writing – review & editing, Data curation.
